# Perspectives of herbs and their natural compounds, and herb formulas on treating diverse diseases through regulating complicated JAK/STAT signaling

**DOI:** 10.3389/fphar.2022.993862

**Published:** 2022-10-17

**Authors:** Jian-Yu Chen, Xiao-Yun Tian, Shan-Shan Wei, Ying-Jie Yang, Shan Deng, Chun-Jie Jiao, Can-Jian Wang, Ke-Dan Chu, Xue-Qin Ma, Wei Xu

**Affiliations:** ^1^ Department of Pharmacology, School of Pharmacy, Fujian University of Traditional Chinese Medicine, Fuzhou, China; ^2^ School of Pharmacy, Second Military Medical University, Shanghai, China; ^3^ Third Affiliated Hospital of Henan University of Traditional Chinese Medicine, ZhengZhou, China; ^4^ Jiangxi University of Chinese Medicine, Nanchang, China; ^5^ Key Laboratory of Hui Ethnic Medicine Modernization, Ministry of Education, Department of Pharmaceutical Analysis, School of Pharmacy, Ningxia Medical University, Yinchuan, China

**Keywords:** natural compounds, herbs, herb formulas, traditional Chinese medicines (TCMs), JAK/STATs

## Abstract

JAK/STAT signaling pathways are closely associated with multiple biological processes involved in cell proliferation, apoptosis, inflammation, differentiation, immune response, and epigenetics. Abnormal activation of the STAT pathway can contribute to disease progressions under various conditions. Moreover, tofacitinib and baricitinib as the JAK/STAT inhibitors have been recently approved by the FDA for rheumatology disease treatment. Therefore, influences on the STAT signaling pathway have potential and perspective approaches for diverse diseases. Chinese herbs in traditional Chinese medicine (TCM), which are widespread throughout China, are the gold resources of China and have been extensively used for treating multiple diseases for thousands of years. However, Chinese herbs and herb formulas are characterized by complicated components, resulting in various targets and pathways in treating diseases, which limits their approval and applications. With the development of chemistry and pharmacology, active ingredients of TCM and herbs and underlying mechanisms have been further identified and confirmed by pharmacists and chemists, which improved, to some extent, awkward limitations, approval, and applications regarding TCM and herbs. In this review, we summarized various herbs, herb formulas, natural compounds, and phytochemicals isolated from herbs that have the potential for regulating multiple biological processes *via* modulation of the JAK/STAT signaling pathway based on the published work. Our study will provide support for revealing TCM, their active compounds that treat diseases, and the underlying mechanism, further improving the rapid spread of TCM to the world.

## Introduction

**FIGURE 1:**
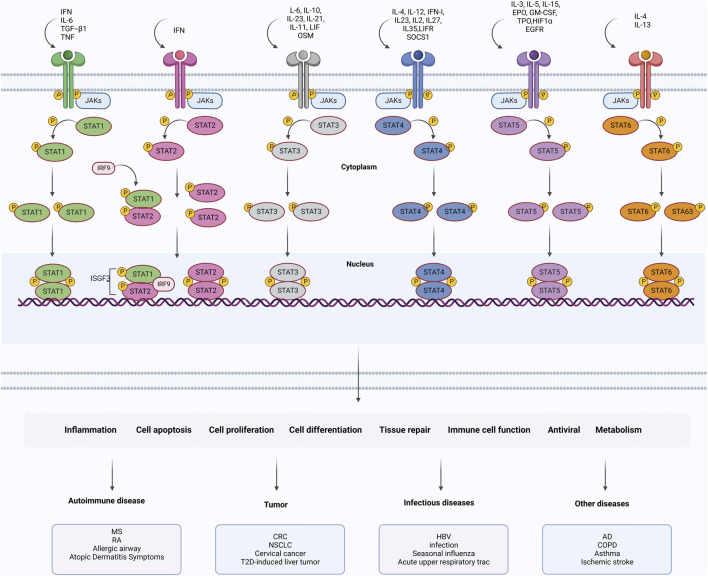
Activation of the JAK/STAT signaling pathway and involved diseases.

Signal transducer and activator of transcriptions (STATs) family members play distinct roles in cell differentiation, tissue repair, and anti-tumor immunity. STAT activation is triggered by Janus kinases (JAKs), which are intracellular tyrosine kinases. Moreover, intracellular tyrosine kinases are activated by the abundance of membrane receptors binding to corresponding cytokines. In response to cytokines binding to specific JAKs, JAKs located in the cytoplasm undergo conformational changes, causing autophosphorylation or transphosphorylation. Subsequently, phospho-JAKs result in docking with different STATs, which causes dimerization of STATs, translocation into the nucleus, and initiate the transcription process (Stark et al., 2018). An overview of the JAK/STAT signaling pathway is presented in Figure 1. JAK/STAT is now recognized as one of the central mediators of widespread and varied human physiological processes. More specifically, its clinical applications have become increasingly important with the discovery of novel clinical syndromes caused by mutations in JAK and STAT genes (Luo et al., 2021a). JAK/STAT signaling regulates many cellular processes essential to maintaining cell homeostasis, whose dysregulation contributes to cancer progressions and inflammatory and autoimmune disorders and COVID-19 emergencies (Chen et al., 2021a; Solimani et al., 2021).

A wide spectrum of diseases has been alleviated by treating with JAK inhibitors. Evidence demonstrates that tofacitinib, a pan-Janus kinase (JAK) inhibitor, is the only FDA-approved drug for RA treatment that downregulated STAT3 signaling mainly by inhibiting JAK expression (Miklossy et al., 2013). Interestingly, even if tofacitinib is the only FDA-approved drug indirectly related to the STAT3 target, it is still more effective than most of these first-line drugs for RA patients, especially in patients with methotrexate-resistant active RA. Additionally, drugs targeting JAK/STAT signaling may effectively assist in the treatment of COVID-19 by restricting cytokine storm syndrome (Luo et al., 2020; Solimani et al., 2021), and three severe COVID-19 cases are reported to be observed to have sustained improvements in outcome with ruxolitinib, which targets the JAK/STAT pathway (Rojas and Sarmiento, 2021).

Chinese herbs, which have widespread distribution throughout China, are the gold resources in China. In the concepts of traditional Chinese medicine (TCM) theory, herbs and herb formulas have been extensively used for treating multiple diseases for thousands of years. Recently, TCM presented a remarkable effectiveness of high epidemic COVID-19 treatment through various approaches (Li et al., 2021a). Therefore, herbs and herb formulas are perspectives for promoting human health against diseases. Despite the wide variety of ingredients in TCM and their complex interactions with the human body, it is still quite difficult to disclose the molecular mechanisms, which severely hampers TCM modernization and internationalization. With the development of chemistry, active ingredients of TCMs and herbs have been further identified by chemists. Afterward, the pharmaceutical effect of active ingredients was subsequently confirmed by pharmacists. Meanwhile, the abundance of potential compounds had been reported to represent biological activities, suggesting that the herb kingdom may be regarded as a gold mine for drug discovery and development (Chen et al., 2022a; Chen et al., 2022b; Liu et al., 2022; Zheng et al., 2022).

Based on the important roles of JAK/STAT signaling in various diseases, in this review, we summarized various TCM formulas, herbs, natural compounds, and phytochemicals which have potential for regulating multiple biological processes *via* modulation of the JAK/STAT signaling pathway based on the published work. Our study will provide support for revealing TCMs, herbs, and their active compounds to treat diseases and the underlying mechanisms.

## Activation of the JAK/STAT signaling pathway and involved diseases

The Janus kinase family consists of four members: JAK1, JAK2, JAK3, and TYK2. All, but JAK3, are ubiquitously expressed, except for JAK3, which is confined to hematopoietic cells. In response to cytokine binding, the receptor of JAKs on the juxta membrane becomes active. Once the receptor is bound, it undergoes intracellular conformation. The STAT family of transcription factors, which includes STAT1, STAT2, STAT3, STAT4, STAT5a, STAT5b, and STAT6, plays distinct roles in cell differentiation, tissue repair, and anti-tumor response. Activation of the JAK/STAT signaling pathways is involved in five processes. First, the cytokines and growth factors engage their corresponding receptors, causing their dimerization and recruitment of related JAKs. Second, the activation of JAK causes tyrosine phosphorylation of the receptors and formation of STAT docking sites. Third, the STAT proteins are phosphorylated by tyrosine. Fourth, the STAT protein dissociates from the receptor to form homodimers or heterodimers. Fifth, in the nucleus, STAT dimers bind DNA and regulate transcription. JAK activation of STAT is the best-characterized pathway, but STATs can also be activated by receptors with intrinsic RTK activity, such as EGFR and PDGFR, and by nonreceptor tyrosine kinases (NRTKs) other than JAKs, such as Src kinase and ABL (Butturini et al., 2020). STATs mediated their biological effects by transactivating a unique profile of target genes dependent on their interactions with STAT-associated regulatory factors. Abnormal activation of JAK/STAT signaling can contribute to disease progressions under various conditions, for example, JAK/STAT signaling regulates inflammation and immunity, cell apoptosis, cell proliferation and differentiation, as well as metabolism, which subsequently cause autoimmune disease, cancer, infectious diseases, and metabolism-related diseases.

## Traditional Chinese medicines or herbs or natural compounds in treating various diseases present good performance both in clinical and pre-clinical conditions

Chinese herbs, which have widespread distribution throughout China, are the gold resources of China. Herbs and herb formulas have been extensively utilized for treating multiple diseases based on traditional Chinese medicine (TCM). It has been reported that TCM presents significant management in treating influenza (Xiong et al., 2020), cardiovascular diseases (Layne and Ferro, 2017), acute pancreatitis (Li et al., 2017), depression (Zhang and Cheng, 2019), and so on. Particularly, in recent years of the COVID-19 outbreak, TCM presents remarkable effectiveness of high epidemic 2019-new coronavirus (SARS-CoV-2) treatment through various approaches (Yang et al., 2020a; Li et al., 2021a). Herbs and herb formulas can be beneficial in promoting human health against diseases. Despite the variety of ingredients in TCM and herbs and their complex interactions with the human body, the molecular mechanisms remain elusive, severely hampering TCM modernization and internationalization. As the development of chemistry has progressed, active ingredients from TCMs and herbs have further been identified by chemists. The pharmaceutical effect of active ingredients was then confirmed by pharmacists. Meanwhile, a vast array of compounds that represent biological activity had been reported, suggesting that the herb kingdom might be considered a gold mine for discovering new drugs, considering the importance of JAK/STAT signaling to various diseases. Hence, we summarized here different TCM formulas, herbs, natural compounds, and phytochemicals with the potential to regulate multiple biological processes via modulation of JAK/STAT signaling. We aimed to identify effective therapies or potential health benefits through regulating STAT agents.

### Chinese herbs and herb formulas, natural compounds, and phytochemicals through regulating the STAT1 signaling pathway

STAT1 signaling is activated by interferon (IFN), interleukin (IL-6), transforming growth factor-1 (TGF-1), tumor necrosis factor (TNF), and angiotensin II (Yu et al., 2009). STAT1 has been predicted as a prognostic biomarker in patients with solid cancer (Zhang et al., 2020), and targeting the IFN/STAT1 pathway could be a promising strategy to protect radioresistance (Liu et al., 2020a). Gemcitabine was identified as an antiviral that induces an IFN response by phosphorylating STAT1 (Li et al., 2020a). Activation of STAT1 signaling is indispensable to various diseases. STAT1, as a mediator of IFN-γ and TLR signaling, participates in endothelial cell dysfunction and cardiovascular diseases (Sikorski et al., 2011). Additionally, IFN-γ-initiated STAT1 promotes Th1 cell differentiation, which was subsets of CD4 effector T cells, and produces IFN-γ, TNF-α, and IL-1β to facilitate β cell apoptosis, finally resulting in progression of type 1 diabetes (Yue et al., 2022). Moreover, Th1 cells and the other subset of CD4 effector T cells, Th17 cells, have been reported to be associated with autoimmune diseases, such as inflammatory bowel disease (IBD), rheumatoid arthritis, and (multiple sclerosis) MS (Delgado-Ramirez et al., 2021). The transcription factor STAT1 binds to and sequences FAS, promoting Th17 cell differentiation and inhibiting Th1 cell development (Meyer Zu Horste et al., 2018). Clonal expansion of antiviral NK cells is regulated by STAT1-mediated epigenetic control of Rsad2 (Wiedemann et al., 2020a). Hence, Chinese herbs and herbal formulas, natural compounds, and phytochemicals for treating diseases and their involved molecular mechanisms regarding STAT1 signaling are summarized to support evidence for their potential health benefits, which is shown in Table 1.

**TABLE 1:** Chinese herbs and herb formulas, natural compounds, and phytochemicals in the treatment of diseases through regulating STAT1.

Candidate	Disease	Signal pathway	Dose/concentration	Animal/cell	Related molecular target		Effect	Reference
					Downregulation	Upregulation		
Baicalin	T2D-induced liver tumor	HKDC1/JAK2/STAT1/Caspase-3	50 mg/L	Glu-cultured HepG2/HuH-7cells	METTL3 and HKDC1	p-JAK2/JAK2, p-STAT1/STAT1, and Caspase-3	Suppressing the migration activity and invasion of HepG2 cells	Jiang et al., (2022)
			20 and 50 mg/kg	BALB/c nude mouse with liver tumor induced by T2D	HKDC1		Inducing liver cell apoptosis and suppressing the development of T2D-induced liver tumors	
Baicalein	Cellular senescence	JAK2/STAT1/NF-κB	0.1, 1, and 10 μM_2_	200 μM H_2_O-induced T98G cells	NF-κB, p-JAK2, p-STAT1, IL-6, IL-8, TNF-α, HGF CXCL1, and MMP-1	BCAT1 and SLC7A5	Increasing viabilities; and delaying senescence	Gao et al. (2021)
Taurine	Allergic rhinitis	IL-35/STAT1	3% w/v	AR mice	p-STAT1, IL-4, IL-5, IL-13	Tregs and IL-35	Normalizing the inflammatory response; reducing AR symptomology; and histopathologic signs of AR	Zhou et al. (2021)
Dihydroartemisinin	MS	STAT1/SOCS3	—	MOG-induced EAE mice		CTLA4, PD-1, SOCS3, and p-STAT1	Reducing spinal cord injury; reducing inflammatory cell infiltration	Du et al. (2021a)
	Gastric cancer	STAT1/KDR/MMP9 P53/BCL2L1/CASP3/7	10, 30 μM	MKN-28 and SGC-7901 cells	p-STAT1, STAT1, p-KDR, KDR, and MMP9	BCL2L1, p27, p53; cleaved-caspase3, caspase-3, and caspase-7	Inhibiting cell proliferation, DNA synthesis, cycle progression, and cell invasion; inducing cell apoptosis	Liang et al. (2021a)
			30 mg/kg	SGC-7901 tumor model in nude mice	Ki-67, BCL2L1, p-KDR, and p-STAT1	CASP3 and P53	Suppressing the tumorigenesis and invasion of gastric cancer	
Diosmetin	Non-alcoholic steatohepatitis	STAT1/CXCL10	320 μM	Palmitic acid stimulated HepG2 cells	LXRα, LXRβ, CHREBP, IL-6, SREBP-1c, p-p65, p-STAT1, and CXCL10		Modulating lipogenesis and inflammatory response	Luo et al. (2021b)
			60 mg/kg	High-fat diet induced mice				
Geniposide	Depression	BTK/JAK2/STAT1	20 μM	LPS-induced BV2 cells	IL-6, TNF-α, CD86, iNOS, p-BTK, p-JAK2, and p-STAT1	BDNF and p-TrkB	Inhibiting inflammatory response	Zheng et al. (2021)
			10 and 40 mg/kg	LPS-induced depressive mice	IL-6, TNF-α, p-BTK/BTK, and p-JAK2/JAK2 p- STAT1/STAT1		Protecting depression	
Kahweol	Skin water loss	STAT1	12.5 μM	HaCaT cells and HEK293T cells		AP-1, NF-kB, CREB, STAT3, STAT1, p-STAT1, HAS2, occludin, and HA	Promoting skin-moisturizing activities	Chen et al. (2021b)
Luteolin	Acute lower respiratory tract infection	MiR-155/SOCS1/STAT1	10 and 50 μM	RSV-induced HEp-2 and A549 cells	SOCS1	MX1, OAS1, ISG15, ISGs, p-STAT1, miR-155	Inhibiting respiratory syncytial virus replication	Wang et al. (2020a)
			50 mg/kg	RSV infected mice			Treating RSV infection	
Rhein	Renal inflammatory injury	lincRNA-Cox2/miR-150–5p/STAT1	10, 20, and 40 μg/ml	Uric acid-induced TCMK-1 cells	IL-6, IL-1β, TNF-α, and STAT1 lincRNA-Cox2	miR-150–5p	Inhibiting renal inflammatory injury of uric acid	Hu et al. (2020)
Polyphenol myricetin	Ischemic cerebrovascular diseases	STAT1	20, 50, and 100 μM	Hypoxia leaded microglia BV2 cells	CD68, iNOS, COX2, STAT1, and p-STAT1		Inhibiting microglia-induced neurotoxicity	Boriero et al. (2021)
TS IIA and AS IV	Lumbar intervertebral disc degeneration	miR-223/JAK2/STAT1	(7, 20, 60 μg/ml) AS IV + (55,160,480 μg/ml) TS IIA	LPS-induced NP cells	miR-223, p-STAT1, and p-JAK2		Inhibiting apoptosis and inflammation	Du et al. (2021b)
TS IIA and Puerarin	IPF	IL6/JAK2/STAT3/STAT1	5 μM Ta IIA +10 μM Pue	NIH-3T3 cell line	JAK2, STAT3, STAT1, and p-STAT1		Inhibiting fibroblast activation and migration	Xue et al. (2021)
			(5 mg/kg) Tan IIA + (14 mg/kg) Pue	BLM-induced IPF mice	IL-6, JAK2, STAT3, STAT1 Col1A1, and MMP9 Col3A1		Alleviating inflammation and pulmonary fibrosis	
GJ-4	VD	JAK2/STAT1	10, 25, and 50 mg/kg	MCAO/R Model rats	NMDAR1, iNOS, COX-2, MMP9, JAK2, and STAT1	SYP and PSD95	Suppressing neuroinflammatory responses; ameliorating the memory dysfunction; and neurological deficits	Liu et al. (2021)
Bisabolane-type sesquiterpenoids isolated from turmeric	Influenza	RIG-1/STAT1,2	25, 50, and 100 μg/ml	A/PR/8/34-induced MDCK, A549 cells	RIG-1, STAT1, p-STAT1, STAT2, p-STAT2, IL-6, IL-10, TNF-α, and IL-8		Reducing inflammation anti-influenza virus	Ti et al. (2021)
Yu-Ping-Feng decoction	NSCLC	STAT1	117 mg	Orthotopic lung tumor-bearing mice	TGF-β and IL-4	IL-2 and IL-12	Prolonging the survival of orthotopic lung tumor-bearing mice; inhibiting LLC cell growth	Zhou et al. (2021)
			1 mg/ml	Macrophage		IL-12, IL-10, iNOS, p-STAT1, and IL-1β	Regulating macrophage polarization to influence the tumor microenvironment	
Yiguanjian decoction	Hepatic fibrosis	STAT1	2 μg/ml	WB-F344 cell line Co-cultured with RAW 264.7 cell line	STAT1, IRF3, IRF5, IRF8, CK19, SOCS3, OV6, SOX9, and EpCAM		Inhibiting macrophage M1 polarization and attenuating hepatic fibrosis	Boriero et al. (2021)
			3.56 mg/kg	2-AAF/CCl4 induced rat	α-SMA, Col (1), TGF-b, CK19, ALT, and AST	CD163		
Sijunzi decoction and Yupingfeng powder	Spleen deficiency	STAT1	2 g/100 g	The spleen deficiency model rat	IL-10, mRNA, IL-6Rα, IL-4, STAT1, STAT4, JAK1	IFN-γ, IL-18, SOCS1, and GATA3	Immuno-enhancement	Xiong and Qian, (2013)
Ganghuo Kanggan decoction	Influenza	RLR	11.6.46.2 g/kg	IAV-infected mice	RIG-I, NF-kB, STAT1, MAVS, IRF-3/IRF-7, and IL-5	IFN-γ, TNF-α, and IL-2	Preventing excessive inflammation	Lai et al. (2020)
Xin-Jia-Xiang-Ru-Yin	Seasonal influenza	IFN-γ/STAT1	14.4 g/kg	FM1-induced IAV mice	IAV, IFN-γ, IRF-1 STAT1, and SOCS1		Reducing the virus replication; ameliorating the pulmonary pathological damage	Li et al. (2018)

Abbreviations; type 2 diabetes (T2D), hexokinase domain containing 1 (HKDC1), idiopathic pulmonary fibrosis (IPF), natural extract from Gardenia jasminoides J. Ellis (GJ-4), N-methyl-D-aspartic acid receptor (NMDAR), Alzheimer’s disease (AD), vascular dementia (VD), influenza A virus (IVA), human influenza virus A/FM1/1/47 (H1N1), multiple sclerosis (MS), myelin oligodendrocyte glycoprotein (MOG), experimental autoimmune encephalomyelitis (EAE), brain-derived neurotrophic factor (BDNF), lipopolysaccharide (LPS), respiratory syncytial virus (RSV), astragaloside IV (AS IV), tanshinone (TS IIA), nucleus pulposus cells (NP, cells), allergic rhinitis (AR), non-small-cell lung cancer (NSCLC), Lewis lung cancer Luciferase cells (LLC-Luc), and 2-acetylamino–fluorene (2-AAF).

Regarding the aspects of Chinese herbal formulas, approximately five herbal formulas regulating STAT1 cascade were collected in preclinical investigational studies, and these were Yu-Ping-Feng decoction (Zhou et al., 2021), Yiguanjian decoction (Boriero et al., 2021), Sijunzi decoction (Xiong and Qian, 2013), Yu-Ping-Feng powder (Xiong and Qian, 2013), Ganghuo Kanggan decoction (Lai et al., 2020), and Xin-Jia-Xiang-Ru-Yin (Li et al., 2018). These formulas, Yiguanjian decoction (Boriero et al., 2021), Sijunzi decoction (Xiong and Qian, 2013), Yupingfeng powder (Lai et al., 2020), Ganghuo Kanggan decoction (Lai et al., 2020), and Xin-Jia-Xiang-Ru-Yin (Li et al., 2018), were crucial for treating hepatic fibrosis, spleen deficiency, and influenza through inhibiting STAT1 signaling, whereas the other formula, Yu-Ping-Feng decoction, induces opposite effects on STAT1 activation to anti-cancer (Zhou et al., 2021). Extracts from two herbs, including a natural extract from Gardenia jasminoides J. Ellis (GJ-4) (Liu et al., 2021) and bisabolane-type sesquiterpenoids isolated from turmeric (Ti et al., 2021), were mentioned here to antagonize Alzheimer’s disease (AD) and infection through inactivating STAT1. Correspondingly, about nine natural compounds and phytochemicals isolated from herbs and plants exhibited good performance in anti-cancer (Liang et al., 2021a; Jiang et al., 2022), treating allergic rhinitis (Zhou et al., 2021), MS (Du et al., 2021a), nonalcoholic steatohepatitis (Luo et al., 2021b), anti-depression (Zheng et al., 2021) and promoting skin-moisturizing activities (Chen et al., 2021b), acute lower respiratory tract infection (Wang et al., 2020a), renal inflammatory injury (Hu et al., 2020), and ischemic cerebrovascular diseases (Boriero et al., 2021) through influencing STAT1 signaling. Natural compounds’ combined utilization has been increasingly popular in recent years for their decrease of deficiencies and complementary advantages. TS IIA and AS IV and TS IIA and Puerarin were presented here for their excellent performance in protecting against lumbar intervertebral disc degeneration (Du et al., 2021b) and idiopathic pulmonary fibrosis (IPF) (Xue et al., 2021) through restricting STAT1 cascade *via* combined utilization. As mentioned above, potential candidates, including baicalin (Jiang et al., 2022), dihydroartemisinin (Du et al., 2021a), kahweol (Chen et al., 2021b), and Yu-Ping-Feng decoction (Zhou et al., 2021), exerted their anti-cancer and anti-infection properties through upregulating cell apoptosis and reinforcing immune system *via* activating STAT1 signaling, which caused high levels of apoptotic proteins, that is, caspases and enhanced the expression of immune cells, such as T cells and B cells. Conversely, compounds effectively treated allergic rhinitis (Zhou et al., 2021) and influenza (Ti et al., 2021) through inhibiting STAT1 signaling, reducing the expression of inflammatory mediators, and regulating cell differentiation.

### Chinese herbs and herb formulas, natural compounds, and phytochemicals through regulating the STAT2 signaling pathway

STAT2 is another member of the STAT family, which is frequently activated in response to type I interferon (IFN α and IFN β) and subsequent JAK1 and TYK2 in the canonical STAT2 cascade (Duncan and Hambleton, 2021). The principal transducer signals of STAT2 is its downstream of the IFN-I and IFN-III receptors by forming a heterotrimeric transcription factor complex containing STAT1 and interferon regulatory factors (IRF9), and the complex is called interferon-stimulated gene factor 3 (ISGF3), which is an interferon-stimulated response element (ISRE) in DNA (Levy et al., 1988). Therefore, deficiency of STAT2 results in impaired STAT1, heterotrimeric formation, prevents the activation of ISGF3, and causes tissue-specific immune defects in response to IFN stimulation (Au-Yeung et al., 2013). Additionally, mutations in STAT2 cause virulent susceptibility and type I interferonopathy in humans (Duncan and Hambleton, 2021). Here, literature regarding the influences of Chinese herbs and herb formulas, natural compounds, and phytochemicals on STAT2 signaling has been collected, which is shown in Table 2.

**TABLE 2:** Chinese herbal formulas or active ingredients in the treatment of infected or inflammatory diseases through influencing STAT2.

Candidate	Disease	Signal pathway	Dose/concentration	Animal/cell	Related molecular targets		Effect	Reference
					Downregulation	Upregulation		
5-HMF	VSV	STAT1–2	10, 25, 50, and 100 μg/ml	VSV induced HEK293T and RAW264.7 cells		IFN-c and IL-4	Promoting lymphocyte proliferative responses	Yuan et al. (2020)
			6 and 12 mg/kg	VSV-induced mice	α-SMA, collagen I, and collagen III	IFIT1, IFIT2, ISG15, Ccl5, IFN-β, p-STAT1, p-STAT2, Ddx58, and RIG-I	Promoting IFN-β production; leading to augmentation of the immune response to VSV infection	Zou et al. (2021)
RAMPtp	IBD	ITSN1-OT1/STAT2	5, 25, and 50 μg/ml	DSS-induced IPEC-J2 cells	p-STAT2, IL-6, TNF-α, and IL-1β		Promoting proliferation and survival of intestinal epithelial cells	Zong et al. (2021)
Euphorbia angustifolia polysaccharide	HBV	JAK/STAT1–2	50, 100, and 200 mg/L	HBV- transfected HepG2 cells	HBsAg and HBeAg	JAK1, STAT1, STAT2, ISGF3, OAS, and PKR	Anti- hepatitis B virus	Mingyu et al. (2019)
			200, 400, and 800 mg/kg	HBV-induced duck	HBsAg and HBeAg		Anti- hepatitis B virus; improving liver injury	
Mahuang tang	Acute upper respiratory tract infection	JAK1/STAT1–2	3.51 g/kg	MNMBD mice		JAK1, p-STAT1, p-STAT2, IgA, IFN-λ, IFNLR1, IRF9, and Mx1	Improving the immune barrier function of mouse nasal mucosa	Xue-mei (2020)
Yinqiao powder			72.54 g/kg					
Si-Ni-San	UC	TBK1/IRF3/STAT1–2	3, 6, and 12 mg/ml	LPS, DMXAA, and Poly (I:C)-induced RAW264.7 cells	ISG15, IFIT1, p-STAT1, Usp18, p-STAT2, and p-IRF3		Suppressing type I IFN responses	Cai et al. (2021)
			0.078, 0.156, and 0.312 g/ml	DSS-induced chronic experimental colitis mouse model	IL-6, IL-12b, IFN-g, IL-17a, Oasl1, Mx2, Usp18, TBK1, and p-TBK1		Improving of chronic experimental colitis model	
SSO and GSLS	NDV	STAT1–2	3, 6, and 12 μg	NDV-induced One-day-old yellow broilers	STAT1, STAT2, PI3K, and IL-6	IFN-c and IL-4	Promoting lymphocyte proliferative responses	Yuan et al. (2020)

Abbreviations; atractylodis macrocephalae polysaccharides (RAMPtp), inflammatory bowel diseases (IBDs), dextran sulfate sodium salt (DSS), hepatitis B virus (HBV), mouse nasal mucosal barrier dysfunction (MNMBD), influenza A virus (IVA), Si-Ni-San (SNS), ulcerative colitis (UC), dextran sulfate sodium salt (DSS), lipopolysaccharide (LPS), Vadimezan (DMXAA), ginseng stem-leaf saponins (GSLS), sunflower seed oil (SSD), Newcastle disease virus (NDV), extracted from the stem and leaf of Panax ginseng C.A., Meyer (E515-D), 5-hydroxymethylfurfural (5-HMF), and vesicular stomatitis virus (VSV).

As a result of the investigational preclinical studies on Chinese herbal formulas, approximately three herbal formulas regulating STAT2 cascades have been identified, including Mahuang tang and Yinqiao powder (Xue-mei, 2020), Si-Ni-San (Cai et al., 2021). In particular, Mahuang tang and Yinqiao powder (Xue-mei, 2020) antagonized acute upper respiratory tract infection by activating STAT2, resulting in improving the immune barrier function of mouse nasal mucosa, whereas, Si-Ni-San inhibited STAT2 activation for alleviating ulcerative colitis (UC) (Cai et al., 2021). Additionally, 5-hydroxymethylfurfural (5-HMF) (Zou et al., 2021) and *Euphorbia angustifolia* polysaccharide (Mingyu et al., 2019) has antiviral potential, including vesicular stomatitis virus (VSV) and hepatitis B virus (HBV), through promoting lymphocyte proliferation and survival (Yuan et al., 2020), whereas *Atractylodis macrocephalus* polysaccharides (RAMPtp), which were extracted from Atractylodis Macrocephale Rhizoma, effectively increased the proliferation and survival of intestinal epithelial cells and maintained the intestinal barrier function, as well as decreased inflammatory cytokines, and finally relieved IBD (Zong et al., 2021). Chinese herbs and herb formulas, natural compounds, and phytochemicals that regulate the STAT2 signaling pathway are shown in Table 2.

### Chinese herbs and herb formulas, natural compounds, and phytochemicals through regulating the STAT3 signaling pathway

Abundant studies have provided substantial insight into the role of T helper 17 (Th17) and regulatory T cells (Tregs) in autoimmune diseases (Dolff et al., 2019; Ding et al., 2021). Th17 cells trigger inflammation by secreting diverse cytokines, which induce other neighboring cells to become activated and, in turn, produce more proinflammatory cytokines and chemokines (Bunte and Beikler, 2019; Kamali et al., 2019; Wu and Wan, 2020). Treg cells, a subgroup of CD4^+^ T cells, are characterized by expressing transcription factor Foxp3 and secreting either TGF-β or IL-10 to counter the Th17-induced inflammation response. STAT3 is another transcription factor in STAT family members that facilitates differentiation of Th17 cells by activating its downstream target genes, retinoic acid receptor-related orphan nuclear receptor (RORγt), and expression of IL-17 (Macaubas et al., 2022). RORγt and IL-17 are unique transcription factors and cytokines of Th17 cells (Chen et al., 2011), which indicated that STAT3 facilitated Th17 cell differentiation and IL-17 expression. Meanwhile, STAT3 restricted Tregs by inhibiting its unique transcription factor Foxp3 (Aqel et al., 2021). Inhibiting STAT3 recalibrates CD4^+^ T responses by interference on both effector and regulatory cells, which provides potential clinically applicable treatment options (Aqel et al., 2021). Additionally, activating STAT3 signals promotes cell proliferation by promoting the progression of the cell cycle and inhibiting apoptosis in cancer (Ashrafizadeh et al., 2021). Blocking STAT3 signaling is considered one of the effective therapeutic options for reversing high self-immune responses *via* antagonizing IL-17A expression, Th17 differentiation, and T lymphocyte proliferation.

Four herbal formulas are summarized here, and they are Buyang Huanwu Decoction (BYHWD), Yanghe decoction (YHD), Maxing Shigan decoction (MXSGD), and Fufang Fanshiliu decoction (FFSLD). Moreover, their distinct effects on STAT3 signaling are shown in Table 3, BYHWD protected against transient ischemic stroke through upregulating p-STAT3. However, the others exhibited good performances for anti-COVID-19, T2DM, and anti-breast cancer by blocking STAT3 signaling. Correspondingly, about nine natural compounds and phytochemicals influence STAT3 signaling for hypertensive heart disease (Ye et al., 2020) and IBD (Zhao et al., 2016). Among all mentioned above, these natural compounds belong to flavonoids (baicalin and quercetin), polyphenols (curcumin, gallic acid, and rosmarinic acid), terpenoids (celastrol and bilobalide), phenylpropanoids (imperatorin), and 3-deoxy-2β,16-dihydroxynagilactone E. Those natural compounds accomplished treatment for myocardial ischemia/reperfusion injury (Xu et al., 2020), diabetic cardiomyopathy (Abdelsamia et al., 2019), IBD (Zhao et al., 2016), alcoholic liver disease (ALD) (Zhu et al., 2017), neuropathic pain (Yang et al., 2021), psoriasis (Zhang et al., 2021a), hypertensive heart disease (Ye et al., 2020), AD (Xiang et al., 2021), cancer cachexia (Chen et al., 2020a), and cancer (Shan et al., 2019). The underlying mechanisms for these compounds treating diseases preferred restraining STAT3 signaling activation. As for synergy utilization of compounds, frankincense and myrrh (Gao et al., 2020), curcumin, and BioPerine (Pillai et al., 2021) were combined applications for multiple myeloma and atherosclerosis through inhibiting STAT3 signaling. The extract of Sophorae Flos (SLE) (Liu et al., 2019), Rosae Multiflorae Fructus, and Lonicerae Japonicae Flos (RLE) decreased STAT3 signaling activation by antagonizing p-STAT3 and resulted in an inhibitory effect on inflammatory cytokines, which finally exerted their effects on RA and melanoma (Liu et al., 2019). Chinese herbal formulas or active ingredients in the treatment of infected or inflammatory diseases through influencing STAT3 are shown in Table 3.

**TABLE 3:** Chinese herbal formulas or active ingredients in the treatment of infected or inflammatory diseases through affecting STAT3.

Candidate	Disease	Signal pathway	Dose/concentration	Animal/cell	Related molecular target		Effect	Reference
					Downregulation	Upregulation		
Baicalin	MI/RI	JAK2/STAT3	20, 60, and 120 mg/kg	(I/R) model rats	iNOS, IL-1β, and IL-6	Arg-1, IL-10, and TGF-β	Alleviating post-I/R myocardial injury; reducing inflammation	Xu et al. (2020)
Curcumin	DCM	Nrf2/HO-1, JAK2/STAT3	100 mg/kg/day	Streptozotocin induced DCM model rats	troponin I, TGF-β1, CK-MB, IL-6, p-JAK2, p-STAT3, and NF-κB	Nrf2 and HO-1	Restoring DCM	Abdelsamia et al. (2019)
	IBD	JAK2/STAT3,6/SOCS	100 mg/kg	2, 4, 6-trinitrobenzene sulfonic acid induced colitis mice	GM-CSF, IL-12p70, IL-15, IL-23, TGF-β1, p-JAK2, p-STAT3, and p-STAT6	IL-4, IL-10, IFN-γ, SOCS1, SOCS3, and PIAS3	Restoring immunologic balance; preventing the chronicity of colitis	Zhao et al. (2016)
Quercetin	ALD	STAT3	4 g/kg	ALD mice	IL- 1β, IL-6, iNOS caspase-3, Bcl-2, p-STAT3, NF-κB, p-Akt, AST, ALT, TBIL, TG, and MDA	SOD, GSH-Px, and IL-10	Preventing alcohol-induced liver injury	Zhu et al. (2017)
Gallic acid	Neuropathic pain	P2X7/NF-κB/STAT3	100 mg/kg	CCI Model rats	P2X7, GFAP, TACE, TNF-α, NF-κB, and p-STAT3		Alleviating mechanical and thermal hyperalgesia; alleviating neuropathic pain	Yang et al. (2021)
Rosmarinic acid	Psoriasis	JAK2/STAT3	15.6–500 μg/m L	LPS-induced HaCat cell line			Inhibiting LPS induced HaCat cells abnormal proliferation	Zhang et al. (2021a)
			40 mg/kg	IMQ-induced mice	IL-23, IL-17A, IL-22, Th17, RoR-γt, STAT3, p-STAT3, and JAK2		Alleviating IMQ-induced psoriasis-like inflammation	
Celastrol	Hypertensive heart disease	STAT3	0.25, 0.5, and 1 nM	Ang II-induced H9C2 cells and rat primary cardiomyocytes	β-MyHC, p-STAT3, Collagen I, and TGF-β1		Attenuating Ang II-induced cardiomyocyte remodeling	Ye et al. (2020)
			0.5 and 1 mg/kg	Ang II and TAC-challenged mice			Preventing Ang II and TAC- associated cardiac injury	
Bilobalide	AD	STAT3	1, 5, and 10 μM	Primary astrocyte	p-STAT3, TNF-α, IL-1β, IL-6, and ROS	NEP, IDE, MMP2, PSD-95, synapsin-1, synaptophysin, and GluR1	Rescuing neuronal deficiency	Xiang et al. (2021)
			0.5 mg/kg	APP/PS1 mice			Reducing amyloid and inflammation	
Imperatorin	Cancer cachexia	STAT3	5, 10, and 20 μM	C2C12 myotube mode	MuRF1, atrogin-1, and C/EBPδ	MyHC	Attenuating myotube atrophy	Chen et al. (2020a)
			25.50 mg/kg	CT26 tumor-bearing mice	MuRF1, atrogin-1, and p-STAT3		Attenuating myotube atrophy; alleviating cancer cachexia	
3-deoxy-2β,16-dihydroxynagilactone E	Cancer	JAK2/STAT3	0.1, 1, 5, and 10 μM	HepG2/STAT3; HepG2/STAT1 cells	P-STAT1, P-STAT3, p-JAK1, p-JAK2, and IL-6	PARP cleavage	Inhibiting the growth of cancer cells	Shan et al. (2019)
Modified citrus pectin	Cardiac hypertrophy	TLR4/JAK/STAT3	100 and 200 mg/kg	ISO-induced rats	ANP, BNP, β-MHC, p-JAK2, p-STAT3, Gal-3, and TLR4	p38	Ameliorating cardiac hypertrophy	Li et al. (2021b)
Frankincense and myrrh	MM	JAK1/STAT3	25, 50, 100, 200, and 400 μg/L	U266 human multiple myeloma cells	VEGF, IL-6, p-JAK1, JAK1, p-STAT3, and STAT3		Inhibiting proliferation of U266 multiple myeloma cells	Gao et al. (2020)
Curcumin and BioPerine	Atherosclerosis	STAT3	150 and 250 μg/ml	THP-1 cell line	CCL2, CD-36, NF-κB, STAT-3, and SCAR-B1		Against atherosclerotic	Pillai et al. (2021)
SLE	Melanoma	STAT3	300 μg/ml	Coculture of B16F10 cells and splenic lymphocytes	IL-6, IL-10, IL-17, TNF-α, and p-STAT3	Th cells, Tc cells, and DCs	Reprograming immune micro-environment; anti-melanoma mechanisms	Liu et al. (2019)
			1.2 g/kg	B16F10 melanomas mice	IL-6, IL-10, IL-17, TNF-α, p-STAT3, MCL1, Bcl-xL, HGF, bFGF VEGF, survivin, cyclin D1, and cyclin D2	Th cells, Tc cells, and DCs		
RLE	RA	JAK2/STAT3	50 and 100 μg/ml	IL-6/sIL-6R-stimulated RA-FLS/PBMCs	RANKL, p-STAT3, p-JAK2, and T IL-1β	OPG	Inhibiting of RANKL production and osteoclasto-genesis	Chen et al. (2020b)
			330 and 660 mg/kg	CIA rats	RANKL, p-STAT3		Alleviating bone erosion; Inhibiting disease progression	
BYHWD	Transient ischemic stroke	PI3K/Akt/Bad; JAK2/STAT3/cyclin d1	50 and 500 μg/ml	OGD/R-induced PC12 cells	cleaved-Caspase-3	p-JAK2, p-STAT3, and cyclin D1	Promoting neurite outgrowth; promoting NSC proliferation	Chen et al. (2020c)
			20 mg/kg	MCAO Model rats		p-EGFR, p-PI3K, Hes1, cyclin D1, p-JAK2, p-STAT3, p-Akt, SOX2, nestin, and p-Bad	Improving the neural stem cell proliferation, astrogenesis, and neurogenesis; inducing the recovery of the neurological functions	
MXSGD	COVID-19	JAK2/STAT3	10, 20, 50, and 100 μg/ml	IL-6 induced RLE-6TN cells	p-JAK2, p-STAT3, Bax, and Caspase 3	Bcl-2	Treating novel coronavirus	Li et al. (2021c)
FFSLD	T2DM	JAK2/STAT3	125, 250, 500, 1000, and 2000 μg/ml	HepG2-IR cells model	ROS, JAK2, STAT3, and Keap-1	p-AKT, Nrf-2, ESR1	Reducing insulin resistance; alleviating T2DM disease	Dai et al. (2022)
YHD	Breast cancer	JAK/STAT1,3	1.43.2.86 g/ml	4T1 breast cancer-bearing mice	MDSCs, iNOS, ARG-1, IL-6, IL-1β, TGF-β, and p-STAT3	IFN-γ, p-STAT1, CD4^+^Tcells, and NKTs	Inhibiting the growth of 4T1 breast tumors; enhancing the antitumor immune response	Mao et al. (2018)

Abbreviations; Buyang Huanwu Decoction (BYHWD), middle cerebral artery occlusion (MCO), oxygen glucose deprivation and reoxygenation (OGD/R), neural stem cells (NSC), dextran sulfate sodium (DSS), ulcerative colitis (UC), isoproterenol (ISO), extract of Sophorae Flos (SLE), chronic constriction injury (CCI), lipopolysaccharide (LPS), imiquimod (IMQ), Alzheimer’s disease (AD), rheumatoid arthritis (RA), collagen-induced arthritis (CIA), extract of Rosae Multiflorae Fructus and Lonicerae Japonicae Flos (RLE), receptor activator for nuclear factor-κB ligand (RANKL), Maxing Shigan decoction (MXSGD), coronavirus disease 2019 (COVID-19), rat lung epithelial type II (RLE-6TN), Fufang Fanshiliu decoction (FFSLD), type 2 diabetes mellitus (T2DM), myocardial ischemia/reperfusion injury (MI/RI), ischemia/reperfusion (I/R), multiple myeloma (MM), diabetic cardiomyopathy (DCM), creatine kinase-MB (CK-MB), The human monocytic cell line (THP-1), 3-deoxy-2β,16-dihydroxynagilactone E, alcoholic liver disease (ALD), B-cell lymphoma (Bcl), inflammatory bowel disease (IBD), Yanghe decoction (YHD), natural killer T cells (NKTs), myeloid-derived suppressor cells (MDSCs), Modified Xiaochaihu Decoction (mXCHD), and chronic hepatitis B (CHB).

### Chinese herbs and herb formulas, natural compounds, and phytochemicals through regulating the STAT4 signaling pathway

As one member of the STAT family involved in transducing signals in response to IL-12, STAT4 plays an integral role in the generation of inflammation during immune responses and immune-mediated diseases, such as rheumatoid arthritis, systemic lupus erythematosus, systemic sclerosis, and psoriasis (Yang et al., 2020b). Activation of the IL-12/JAK/STAT4 cascade produces an abundance of IFN-γ and promotes Th1 cell differentiation by inducing an ETS transcription factor, EMR (Ouyang et al., 1999). IL-12 facilitated interferon regulatory factor-1 (IRF1) in natural killer (NK) and T cells (Galon et al., 1999), and it also increased the expression of IRF4 and IRF8 genes, resulting in intensive innate immune responses (Lehtonen et al., 2003). Additionally, STAT4 gene polymorphisms are at a higher risk of type 2 diabetes and systemic sclerosis (Xu et al., 2016), and STAT4 was regarded as diagnostic biomarker candidates and therapeutic targets for heart failure combined with depression (Huang et al., 2022). Based on its essential role in inflammation and autoimmunity, it is a promising therapeutic target for autoimmune diseases.

As a result of the investigational preclinical studies on Chinese herbal formulas, approximately two herbal formulas regulating STAT4 cascades have been identified, namely, Liuweibuqi capsules and Hei-Gu-Teng-Zhui-Feng-Huo-Luo granule. Liuweibuqi capsules were reported to be effective in treating chronic obstructive pulmonary disease (COPD) through antagonizing STAT4, but activating STAT6 (Shen et al., 2019). The Hei-Gu-Teng-Zhui-Feng-Huo-Luo granule downregulated IL-12-stimulated STAT4 signaling and caused low levels of TNF-α, IL-1β, and IL-6 in collagen-induced arthritis (Zheng et al., 2018). Three kinds of extracts from herbs, namely, Pinellia pedatisecta Schott extract (PE) (Wang et al., 2021a), aqueous extract of Fritillariae cirrhosae (FC-AE) (Li et al., 2020b), and Euphorbia helioscopia L. aqueous extract (EAE) exhibited their excellent performance in alleviation of COPD and anti-cancer through inhibiting STAT4 signaling. Meanwhile, Euphorbia helioscopia L. aqueous extract (EAE) (Ling-ling et al., 2021) represented its therapeutical effect on COPD through activating STAT6 cascades (Ling-ling et al., 2021). Four compounds isolated from herbs, saikosaponin A, cynaropicrin, xanthatin, and baicalin, were reported here for breast cancer (Zhao et al., 2019), colorectal cancer (CRC) (Zheng et al., 2020), Non-small-cell lung cancer (NSCLC) (Zheng et al., 2020), and sepsis (Liu et al., 2020b). To be specific, saikosaponin A inhibited breast cancer growth and promoted a shift of Th1/Th2 balance toward Th1 through activating STAT4 signaling (Zhao et al., 2019), which presented anti-cancer performance. Xanthatin antagonized NSCLC through inhibiting STAT4 signaling and suppressing cell proliferation, migration, and invasion (Zhang et al., 2021b). Cynaropicrin and baicalin inhibited both STAT3 and STAT4 signaling, resulting in frequent apoptosis and Treg differentiation (Liu et al., 2020b; Zheng et al., 2020). Chinese herbal formula or active ingredients in the treatment of infected or inflammatory diseases through influencing STAT3 are shown in Table 4.

**TABLE 4:** Chinese herbs and herb formulas, natural compounds, and phytochemicals in the treatment of diseases through mediating STAT4.

Candidate	Disease	Signal pathway	Dose/concentration	Animal/cell/patient	Related molecular target		Effect	Reference
					Downregulation	Upregulation		
Saikosaponin A	Breast cancer	IL-12/STAT4	100 mg/kg	Breast cancer in rats	IL-4, IL-10, and Th2	IFN-γ, IL-12, Th1, and p-STAT4	Inhibiting breast cancer growth; shifting Th1/Th2 balance toward Th1	Zhao et al. (2019)
Cynaropicrin	CRC	LIFR/STAT3 (STAT3-STAT4)	2.5, 5, and 7.5 μM	Human CRC cell lines (HCT116, RKO, and DLD-1)	LIFR, p-STAT3, and STAT3/STAT4	Cl-PARP1, Bax, and Bcl-2	Inducing apoptosis; inducing loss of migration potential	Zheng et al. (2020)
			2.5 and 5 mg/kg	HCT116 cells-bearing nude mice	LIFR and p-STAT3	Cl-PARP1, Bax, and Bcl-2	Inhibiting the Growth of CRC	
Xanthatin	NSCLC	JAK2/STAT4	1.5 and 4.5 μmol/L	A549 cells	BARD1, p-JAK2, and p-STAT4		Suppressing cell proliferation, migration, and invasion	Zhang et al. (2021b)
			1 and 3 μmol/L	H1299 cells				
Baicalin	Sepsis	RhoA-ROCK STAT3,4,5	100, 200, and 300 mg/kg	CLP-induced sepsis model mice	Th1, Th17, T-box, ROCK1, p-STAT4, p-STAT3, IFN-γ, IL-17, RORγt, and RhoA	Treg, Foxp3, IL-10, and p-STAT5	Ameliorating sepsis-associated pancreatic injury; regulating Th1, Th17, and Treg responses	Liu et al. (2020b)
PE	Cervical cancer	SOCS1/JAK2/STAT1,4,5	12.5, 25, 50, and 100 μg/mL	TADCs	SOCS1	p-JAK2/JAK2, p-STAT1/STAT1, p-STAT4/STAT4, p-STAT5/STAT5, CD80, CD86, IL-12, and p70	Restoring the function of cervical TIDCs to elicit antitumor CTL responses	Wang et al. (2021a)
FC-AE	Tumors	STAT1, STAT4	37.62 μg/ml	A549 cells	Bcl-2	Caspase-3, Bax, IL-12, IFN γ, STAT1, and STAT4	Mediating apoptosis; modulating immune responses	Li et al. (2020b)
			2.5 mg/ml (0.2 ml)	Xenograft model mice				
EAE	COPD	IL-12/STAT4; IL-4/STAT6	1.25, 2.5, and 5 g/kg	Cigarette and LPS-induced COPD rats	GGT, IL-1β, IL-12, IL-17A, TNF-α, MMP-2/TIMP-2, and STAT4	MMP-9/TIMP-1, IL-4, and STAT6	Improving the parameters of pulmonary function; alleviating the pathological structure of lung tissue	Ling-ling et al. (2021)
Liuweibuqi capsules	COPD	STAT4/STAT6	3, 9, and 27 capsules/d	COPD patients	STAT4, MMP-9, IFN-γ, nd IL-6	STAT6, TIMP-1, and IL-4	Reducing inflammatory responses; improving pulmonary function of people suffering from stable COPD	Shen et al. (2019)
Hei-Gu-Teng-Zhui-Feng-Huo-Luo granule	RA	IL-12/STAT4	6.25, 12.5, 25, 50, 100, and 200 nM	LPS-induced U937 cells	TNF-α, IL-1β, and IL-6		Inhibiting inflammatory response	Zheng et al. (2018)
			—	CIA mice	TNF-α, IL-1β, IL-6, IL-12, and STAT4			

Abbreviations; colorectal cancer (CRC), leukemia inhibitory factor receptor (LIFR), poly (ADP-ribose), polymerase 1 (PARP1), non-small-cell lung cancer (NSCLC), homosapiens BRCA1 associated RING, domain 1(BARD1), Ras homolog gene family, member A (RHoA), Rho-associated kinase (ROCK), cecal ligation and puncture (CLP), retinoic acid receptor-related orphan receptor γt (RORγt), forkhead/winged helix transcription factor (Foxp3), tumor-associated dendritic cells (TADCs), cytotoxic T lymphocytes (CTL), Pinellia pedatisecta Schott extract (PE), aqueous extract of Fritillariae cirrhosae (FC-AE), Euphorbia helioscopia L. aqueous extract (EAE), chronic obstructive pulmonary disease (COPD), matrix metalloprotein (MMP), tissue inhibitor of metalloproteinase (TIMP), rheumatoid arthritis (RA), and collagen-induced arthritis (CIA).

### Chinese herbs and herb formulas, natural compounds, and phytochemicals through regulating the STAT5 signaling pathway

Two adjacent genes encoded STAT5 in mammals, namely, Stat5a and Stat5b, which drive tumor development, metastasis, survival and drug resistance to treatment (Verhoeven et al., 2020), NK cell development, maturation, survival, and cytotoxicity (Wiedemann et al., 2020b). JAK/STAT5 is frequently activated by IL-2, IL-15, EPO, GM-CSF, TPO, and other factors (Rani and Murphy, 2016; Orlova et al., 2019). Different activation procedures of JAK/STAT5 induced by different cytokines cause distinct actions. IL-2 and IL-15 at upstream of STAT5 promote the early and late stages of the adaptive NK cell response to different mouse cytomegalovirus (MCMV) infections (Wiedemann et al., 2020b). GM-CSF effectively drives the expression of inflammatory factors in macrophages through activating the STAT5 cascade, which upregulated the immune stimulatory gene levels, and in contrast to tissue remodeling factors increased caused by loss of STAT5 (Jesser et al., 2021). Moreover, lessened pathogenic Th17 cells and monocyte-derived cells (MDCs) in the meninges were observed in the STAT5 tetramer-deficient Stat5a–Stat5b N-domain double knock-in mouse strain, suggesting that the GM-CSF-STAT5 tetramer-CCL17 cascade in MDCs promotes autoimmune neuroinflammation (Monaghan et al., 2021). IL-2/STAT5 signaling contributed to monitoring the ratio of Th9/Th17-like *in vitro* and allergic disease. To be specific, STAT5 restricted a Th17-like program in the process of Th9 cell differentiation (Canaria et al., 2021). As shown above, STAT5 regulates the development of inflammation and tumor-related diseases (Surbek et al., 2021).

In total, eight compounds were collected for their biological potential for treating diverse diseases. Dihydromyricetin blocked STAT5 signaling and reduced oxidative stress, mitigating mast cell activity, which effectively alleviated allergic diseases (Chang et al., 2021). Upregulation of cryptotanshinone on the expression of p-STAT5 supported protection against ischemic stroke (Zhu et al., 2021), whereas the inhibitory effect of cryptotanshinone effectively antagonized chronic myelocytic leukemia (CML) (Dong et al., 2018). Additionally, tetrahydroquinoline derivatives (Polomski et al., 2021) and taxodione (Uchihara et al., 2018) antagonized chronic myelocytic leukemia by inhibiting activation of STAT5 signaling. Moreover, silibinin (Rugamba et al., 2021), naringenin (Niu et al., 2018), xanthoplanine (Shi et al., 2020), and menthol (Suzuki et al., 2020) exhibited the biological potential of NSCLC, autoimmune inflammatory disorders, and arterial inflammation through inhibiting STAT5 signaling. Extracts of two herbs are mentioned here to antagonize diverse diseases through regulating STAT5. Ginseng powder possesses the potential for immunity upregulation through increasing activation of B-cell, Th1 cell, and NK-cell *via* both activating STAT5 signaling and blocking STAT3 signaling (Park et al., 2022). *Trifolium repens* Linn (Sarno et al., 2020) was reported to be effective in treatment of chronic myelogenous leukemia through inhibiting p-STAT5. Bu-Shen-Zhu-Yun decoction (Feng et al., 2021) and Bufei Yishen formula (Zhao et al., 2018) are crucial for treating hyperprolactinemia infertility and COPD through activating STAT5 signaling. You-Gui-Yin (Li et al., 2020c) and Guizhi-Shaoyao-Zhimu decoction (Zhang et al., 2019) were effective in the treatment of chronic kidney disease and rheumatoid arthritis through inhibiting STAT5 signaling, which shown in Table 5.

**TABLE 5:** Chinese herbs and herb formulas, natural compounds, and phytochemicals in the treatment of diseases through regulating STAT5.

Candidate	Disease	Signal pathway	Dose/concentration	Animal/cell	Related molecular target		Effect	Reference
					Downregulation	Upregulation		
Dihydromyricetin	Allergic diseases	STAT5	10 and 100 µM	DNP-IgE-induced KU812 cells	ROS, TNF-α, IL-6, p38, MAPK, and p-STAT5		Reducing the oxidative stress; mitigating the mast cell activity	Chang et al. (2021)
Cryptotanshinone	Ischemic stroke	STAT5	15 mg/kg	MCAO model rats		Foxp3, p-STAT5, and IL-2	Attenuating the infarct region in the MCAO model; improving ischemic stroke	Zhu et al. (2021)
	CML	STAT3 and STAT5	0.1, 1, 10, 30, and 50 μmol/L	K562 cells and K562/ADR	c-Myc, p-STAT5, p-STAT3, MRP, STAT3, nd P-gp		Suppressing both onco-proliferative and drug-resistant pathways in CML cells	Dong et al. (2018)
Tetrahydroquinoline derivatives	CML	STAT5	0.1, 1, 5, and 10 µM	KU812 and MV4-11 cells	p-STAT5 and STAT5		Inducing apoptosis in both leukemic cell lines	Polomski et al. (2021)
Taxodione	CML	STAT5	10 and 100 µM	K562 cells	MRC complex III, MRC complex V, p210^BCR−ABL^, c-Myc, p-STAT5, STAT5, and Akt	ROS and Bim	Inducing apoptotic cell death in K562 cells	Uchihara et al. (2018)
Silibinin	NSCLC	EGFR/JAK2/STAT5	50 and 100 µM	A549, H460, and H292 cells	CDK4, cyclin D1, cyclin E, MMP9, MMP2, SOX2, OCT4, NANOG, PD-L1, p-EGFR, p-JAK2, p-STAT5, and p-Akt	p21 and p27	Inhibiting NSCLC cell migration and invasion; inducing G0/G1 phase cell cycle arrest and apoptosis; inhibiting tumor sphere formation	Rugamba et al. (2021)
				HUVECs	p-EGFR, p-STAT5, and p-Akt		Inhibiting tumor angiogenesis and invasion	
Naringenin	Autoimmune inflammatory disorders	IL-2/IL-2R/STAT5	20, 40, and 80 µM	anti-CD3/CD28-induced lymph node cells	TNF-α, IL-4, IL-6, IL-17A, IFN-γ, and p-STAT5		Inhibiting T cell proliferation and secretion of cytokines	Niu et al. (2018)
				Lymph node cells from MOG-induced EAE mice	IL-2, IL-4, IL-6, IL-17A, IFN-γ, and p-STAT5			
Xanthoplanine	Arterial inflammation	CrkL-STAT5	50 and 100 µM	LPS (100 ng/ml) and IFN-γ (20 ng/ml)-induced mouse peritoneal macrophages	ROS, p-STAT5, CrkL-STAT5, TNF-α, IL-6, IL-12, MDA, and SOD	IL-10, TGF-β1, and Arg-1	Attenuating macrophage polarization toward M1 phenotype to reduce inflammatory response	Shi et al. (2020)
Menthol	—	STAT5	0.1, 0.33, and 1 mM	Lactating MECs	β-casein, STAT5, p-STAT5, mTOR, CNN3, Lalba, and <α-lactalbumin, Glut1	Cytoplasmic lipid droplets	Suppressing milk production of lactating MECs	Suzuki et al. (2020)
Ginseng powder	—	STAT5	100 μg/ml	NK-92MI, K562 cells	FasL, IFN-γ, p-STAT3	GZMB, p-Akt, Akt, STAT5, and p-STAT5	Inducing B-cell activation, Th1-type T-cell activation, and NK-cell activation	Park et al. (2022)
			100 and 200 mg/kg	5-week-old female C57BL/6 mice				
*Trifolium repens*	Chronic myelogenous leukemia	BCR-ABL/STAT5	0.5 and 1 mg/ml	K562 cells	p-STAT5, p-AKT, and p-p38		Reducing survival of K562 cells	Sarno et al. (2020)
Bu-Shen-Zhu-Yun decoction	Hyperprolactinemia infertility	JAK2/STAT5	10 mM	Prolactin induced GT1-7 cells		p-JAK2, p-STAT5, PRLR, kisspeptin. CSN5, GATA1	Inducing the deubiquitination of PRLR; alleviating the clinic manifestations of hyperprolactinemia infertility	Feng et al. (2021)
Bufei Yishen formula	COPD	STAT3; STAT5	2.2, 4.4, and 8.8 g/kg	Cigarette smoke- and bacterial infection-induced COPD rats	IL-1β, TNF-a, IL-6, IL-17A, RORγt, and p-STAT3	IL-10, Foxp3, and p-STAT5	Improving the lung functions; attenuating inflammatory conditions	Zhao et al. (2018)
You-Gui-Yin	Chronic kidney disease	HIF1α/STAT5	10, 20, and 40 g/kg	Adenine- induced CKD rats	p-EPOR/EPOR, p-JAK2/JAK2, and p-STAT5/STAT5	ACTH, CORT, EPO, T3, T4, EPO/SEPOR, LH, FSH, DHT, E2, and HIF1α	Improving CKD and its associated low reproductive function	Li et al. (2020c)
Guizhi-Shaoyao-Zhimu decoction	Rheumatoid arthritis	STAT5	0.4, 0.8, and 1.6 mg/ml	TNF-α induced MH7A cells	Bcl-2, JAK2, STAT3, STAT5 IL-6, IL-8, MMP1, MMP2, MMP3	Caspase3, Bax, Caspase 9, SOCS1	Suppressing inflammatory responses; inhibiting invasion and migration of synovial fibroblasts; and inducing apoptosis in synovial fibroblasts	Zhang et al. (2019)
			0.8, 1.6, and 3.2 mg/ml	LPS stimulated RAW264.7 cells	TNF-α and IL-1β			Zhang et al. (2019)
			800, 1600, and 3200 mg/kg	CIA rats	TNF-α, IL-1β, IL-6, and IL-17A			

Abbreviations; dinitrophenyl (DNP), middle cerebral artery occlusion (MCAO), forkhead box protein P3 (FOXP3), chronic myelocytic leukemia (CML), P-glycoprotein (P-gp), mitochondrial respiratory chain (MRC), Non-small-cell lung cancer (NSCLC), myelin oligodendrocyte glycoprotein (MOG), malondialdehyde (MDA), superoxide dismutase (SOD), whey acidic protein (WAP), mammary epithelial cells (MECs), calponin 3 (CNN3), sterol regulatory element-binding transcription factor 1 (SREBF1), α-casein (Csn1), β-casein (Csn2), κ-casein (Csn3), alpha-lactalbumin (LALBA), Fas ligand (FasL), granzyme B (GzmB), prolactin (PRL), estradiol (E2), growth hormone (GH), cytokine-inducible SH2-containing protein (CISH), prolactin receptor (PRLR), cyclin-dependent kinase (CDK), erythropoietin (EPO), erythropoietin receptor (EPOR), adreno-cortico-tropic-hormone (ACTH), corticosterone (CORT), the soluble form of the erythropoietin receptor (SEPOR), type II, and collagen-induced arthritis (CIA).

### Chinese herbs and herb formulas, natural compounds, and phytochemicals through regulating the STAT6 signaling pathway

The STAT6, another important member of the STAT family, is activated primarily by IL-4 and IL-13 (Czimmerer et al., 2018). STAT6 has been reported to be crucial in Th2 cell differentiation (Forbes et al., 2010). Additionally, variants of STAT6 were closely associated with asthma risk (Qian et al., 2014), and deficiency of STAT6 affects immune function, glycolysis, and B cell morphology, which contributes to various diseases. Therefore, regulation of B cells by STAT6 can perhaps be a prospective therapeutic approach for human diseases (Wang et al., 2021b). As shown in Table 6, two Chinese herbal formulas regulating STAT6 cascades have been identified, namely, Srolo Bzhtang (Jing et al., 2019) and Banhahubak-tang tablet (Nam et al., 2020), which both antagonized airway inflammation (Jing et al., 2019) and allergic asthma (Nam et al., 2020) through blocked STAT6 signaling. Total glucosides of paeony blocked STAT6 signaling and promoted M2 macrophage polarization for treating lupus nephritis (Forbes et al., 2010). Sixteen compounds isolated from herbs or plants presented their biological activities for treating diseases. Three compounds, namely, ginsenoside Rb1 (Zhang et al., 2018), luteolin (Wang et al., 2020b), and physalin D (Ding et al., 2019) exhibited potential therapeutic effects on coronary artery disease, inflammation, and imbalance of macrophage polarization-related diseases. The others preferentially executed their primary role for downregulation of STAT6 signaling in the treatment of various diseases, for instance, atopic dermatitis symptoms (Sung and Kim, 2018), cancer metastasis (Yang et al., 2018), allergic airway inflammation (Chen et al., 2018; Li et al., 2020d), asthma (Ma et al., 2020), allergic asthma (Chen et al., 2017; Ma et al., 2019), diabetes-associated inflammation (Oh et al., 2020), chronic kidney disease (Li et al., 2020e), cervical cancer (Duan et al., 2021), inflammation (Liu et al., 2018), insulin resistance (Subash-Babu and Alshatwi, 2018), and AD (Choi et al., 2020).

**TABLE 6:** Chinese herbs and herb formulas, natural compounds, and phytochemicals in the treatment of diseases through regulating STAT6.

Candidate	Disease	Signal pathway	Dose/concentration	Animal/cell	Related molecular target		Effect	Reference
					Downregulation	Upregulation		
Crocin	Atopic dermatitis symptoms	NF-κB, STAT6	(0.1%,0.3%) 100 mg	DfE-induced AD model mice	p-STAT6/STAT6, p-IκBα, IL-4, IL-5, IL-13, IgE, and TARC		Ameliorating atopic dermatitis symptom	Sung and Kim, (2018)
Celastrol	Cancer metastasis	STAT6	31.2.62.5 and 125 nM	IL-13 induced RAW264.7	MRC1, Arg1, Fizz1, Mgl2, CD11c, and p-STAT6		Inhibiting the M2-like polarization of macrophages	Yang et al. (2018)
			10 mg/kg	4T1 tumor cells-bearing mice			Inhibiting lung metastasis of breast cancer	
Tetrahydro curcumin	Allergic airway inflammation	IL4Ra/JAK1/ST Notch1/Notch2	120 mg/kg	OVA-induced mice	MDA, IL-13, IL-4, IL-5, Th17, GATA3, Tc2, Tc17, IL-4Rα, p-JAK1/JAK1, p-STA6/STAT6, Jagged1, Jagged2, NICD1/Notch1, and NICD2/Notch2	GSH	Relieving airway inflammation	Chen et al. (2018)
Ginsenoside Rb1	Coronary artery disease	IL-4 and IL-13/STAT6	20 μM	LPS-stimulated peritoneal macrophages	MMP-9	IL-10, IL-4, IL-13, and p-STAT6/STAT6	Promoting M2 macrophage polarization	Zhang et al. (2018)
			50 mg/kg	ApoE^−/−^ mice	MMP-9	SMCs	Increasing plaque stability	
Berberine	Asthma	STAT6	1 μM	IL-4 and TNF-α-stimulated BEAS-2B cells	IL-6, CCL11, and STAT6		Relieving airway inflammation	Ma et al. (2020)
OODBL	Allergic asthma	JAK3/STAT6	5 mg/ml	IL-4- stimulated A549 cells	p-JAK3, p-STAT6, eotaxin-1, ALOX15	p-Akt	Preventing and treating allergic diseases	Chen et al. (2017)
Asaronic acid	Diabetes-associated inflammation	IL-4Rα/Tyk2/STAT6; GLUT1/Akt/Mtor/AMPK	1,10,20 μM	IL-4- stimulated J774A.1 cells	p-Tyk2, p-STAT6, p-Akt, p-mTOR, p-AMPKα, IL4Rα, arginase-1, GLUT1, COX-2, CTGF, α-SMA, SR-A, SR-B1, and ABCG1	VEGF, PDGF, IL-10, and PPARγ	Inhibiting diabetic macrophage dysfunction due to M2 activation	Oh et al. (2020)
Bixin	Chronic kidney disease	STAT6	40 μM	IL-4-induced HK2 cells	ECM, P62, p-STAT6		Improving partial EMT of tubular cells and renal interstitial fibrosis	Li et al. (2020e)
			100 mg/kg	UUO model mice				
Cyanidin-3-O-β-glucoside	Allergic asthma	IL-4Rα-STAT6	400 mg/kg	OVA-induced BALB/c mice	IL-4, IL-5, IL-13, IL-4Rα, p-JAK1, and p-STAT6		Alleviating allergic airway inflammation	Ma et al. (2019)
Lanatoside C	Cervical cancer	JAK2/STAT6/SOCS2	0.5, 1, and 2.5 μM	HeLa and BEAS-2B cells	MMP, p-JAK2, and p-STAT6	cleaved Caspase-9, cleaved Caspase-3, cleaved PARP, p21, cyclin B1, and SOCS2	Inhibiting cell proliferation, migration and inducing apoptosis	Duan et al. (2021)
Luteolin	Inflammation	STAT3 and STAT6	0.5, 10, 20, 40, and 80 μM	LPS and IFN-γ-induced RAW264.7 cells	p-STAT3, iNOS, IL-1β, IL-6, and TNF-α	Arg1, IL-10, CD206, CD163, IL-13, and p-STAT6	Releasing anti-inflammatory factors; alleviating inflammation	Wang et al. (2020b)
Mahonia oiwakensis Hayata	Inflammation	STAT1 and STAT6	150 μg/ml	LPS-induced RAW264.7 cells	NO, MCP-1, RANTES, TNF-α, p-STAT6, p-STAT1, and p-NF-κB	VEGF	Promoting anti-inflammation VEGF-mediated tissue remodeling	Liu et al. (2018)
Ononitol monohydrate	Insulin resistance	STAT6	1.6 and 3.2 μM	HMSC and PBMNCs	leptin, C/EBPα, TB4R, C/EBPα, PPARγ, fas, LPL, IL-4, p-STAT6, TNF-α, and ap2	UCP-1, PRDM16, PPARγC1α, and SREBP-1c	Enhancing browning of maturing adipocyte and glucose homeostasis via insulin sensitivity	Subash-Babu and Alshatwi, (2018)
Physalin D	Imbalance of macrophage polarization	STAT1/6	10, 30, and 50 μM	BMMs	p-STAT1 and iNOS	p-STAT6 and Arg1	Regulating macrophage M1/M2 polarization	Ding et al. (2019)
Piperine	AD	STAT6/GATA3/IL-4	2 and 4 mg/ml	TMA-induced AD-like mouse model	IL-1β, TNF-α, IL-4, p-STAT6/STAT6, IgE, and GATA3		Improving AD symptoms	Choi et al. (2020)
Protocatechuic acid	Allergic airway inflammation	IL-4Rα/STAT6; Jagged 1/Jagged2–Notch1/Notch2	50 mg/kg	OVA-induced mice	IL-4, IL-5, IL-13, GATA3, IL-4Rα, p-STAT6, p-JAK1, Jagged1, Jagged2, NICD1, NICD2, Notch1, and Notch2		Preventing allergic asthma	Li et al. (2020d)
Total glueosides of paeony	Lupus nephritis	IL-4/STAT6/PD-L2	20 and 40 ug/ml	IL-4 or LPS plus IFN-g-induced Raw264.7 cell lines	p-STAT1	F4/80^+^CD11b^+^ CD206^+^ M2, PD-L1, PD-L2, and p-STAT6	Promoting the M2 macrophage polarization	Liang et al. (2021b)
Srolo Bzhtang	Airway inflammation	IL-13/STAT6	1.67 and 2.50.3.34 g/kg	CS-induced-CB model	IL-13, p-STAT6, and MUC5AC	PD-L1, PD-L2, and p-STAT6	Ameliorating airway inflammation	Jing et al. (2019)
			100 and 200 mg/kg	PBS-induced murine	p-STAT1		Ameliorating the renal inflammation and injury in lupus nephritis	
Banhahubak-tang tablet	Allergic asthma	JAK1/STAT6	0.1, 1, 10, and 100 μg/ml	PM10-T reated A549	p-JAK1/JAK1, p-STAT6/STAT6, IgE, IgG, IL-4, IL-5, IL-13, TNF-α, IL-1β, IL-6, IL-8, and IL-17A		Suppressing airway inflammation, mucus hypersecretion, and airway remodeling	Nam et al. (2020)
			6.29.62.9 and 629 mg/kg	OVA and PM10-Induced Mice				

Abbreviations; dermatophagoides farinae extract (DfE), atopic dermatitis (AD), thymus and activation-regulated chemokine (TARC), mannose receptor C-type 1 (MRC1), arginase 1 (Arg1), resistin-like molecule alpha1 (Fizz1), ovalbumin (OVA), glutathione (GSH), lipopolysaccharide (LPS), smooth muscle cells (SMCs), 1,6-O, O-diacetylbritannilactone (OODBL), arachidonate15-lipoxygenase-1 (ALOX15), glucose transporter type 1 (GLUT1), connective tissue growth factor (CTGF), scavenger receptor type A (SR-A), scavenger receptor class B type I (SR-B1), ATP, binding cassette subfamily G member 1 (ABCG1), vascular endothelial growth factor (VEGF), platelet-derived growth factor (PDGF), peroxisome proliferator-activated receptor γ (PPARγ), unilateral ureteral obstruction (UUO), extracellular matrix (ECM), epithelial–mesenchymal transition (EMT), mitochondrial membrane potential (McMP), poly ADP-ribose polymerase (PARP), regulated upon activation, normal T cell expressed, and secreted (RANTES), lipoprteinlipase (LPL), uncoupling protein 1 (UCP-1), PR, domain-containing protein 16 (PRDM16), sterol regulatory element-binding protein-1c (SREBP-1c), bone marrow macrophages (BMMs), Notch intracellular domain (NICD), cigarette smoke (CS), chronic bronchitis (CB), and Mucin-5AC (MUC5AC).

## Summary and prospect

JAK/STAT signaling pathways are closely implicated in multiple biological processes involved in cell proliferation, apoptosis, inflammation, differentiation, immune response, and epigenetics (Chen et al., 2021c). In response to extracellular signaling proteins, such as IL-6, TNF-α, and IFNs, which are widespread in an inflammatory microenvironment, JAK/STAT pathways are then activated. Activation of JAK/STAT signaling either contributes to direct expression of the proinflammatory cytokine or induces inflammatory cell proliferation, promoting secretion of cytokines, where both assist in forming a positive feedback loop with JAK/STAT and result in more disease severity. Moreover, multitudinous cascades have been reported to represent complicated crosstalk with JAK/STAT pathways through diverse cytokine pathways. For example, activation of NF-κB leads to increase of inflammatory cytokines, such as IL-6, which result in STAT3 activation and transcription; produces various cytokines; and in turn, promotes NF-κB and STAT3 activation (Grivennikov et al., 2010). Notch-driven differentiation of astrocytes induced by STAT3 is essential in the development of the central nervous system (Kamakura et al., 2004).

**FIGURE 2:**
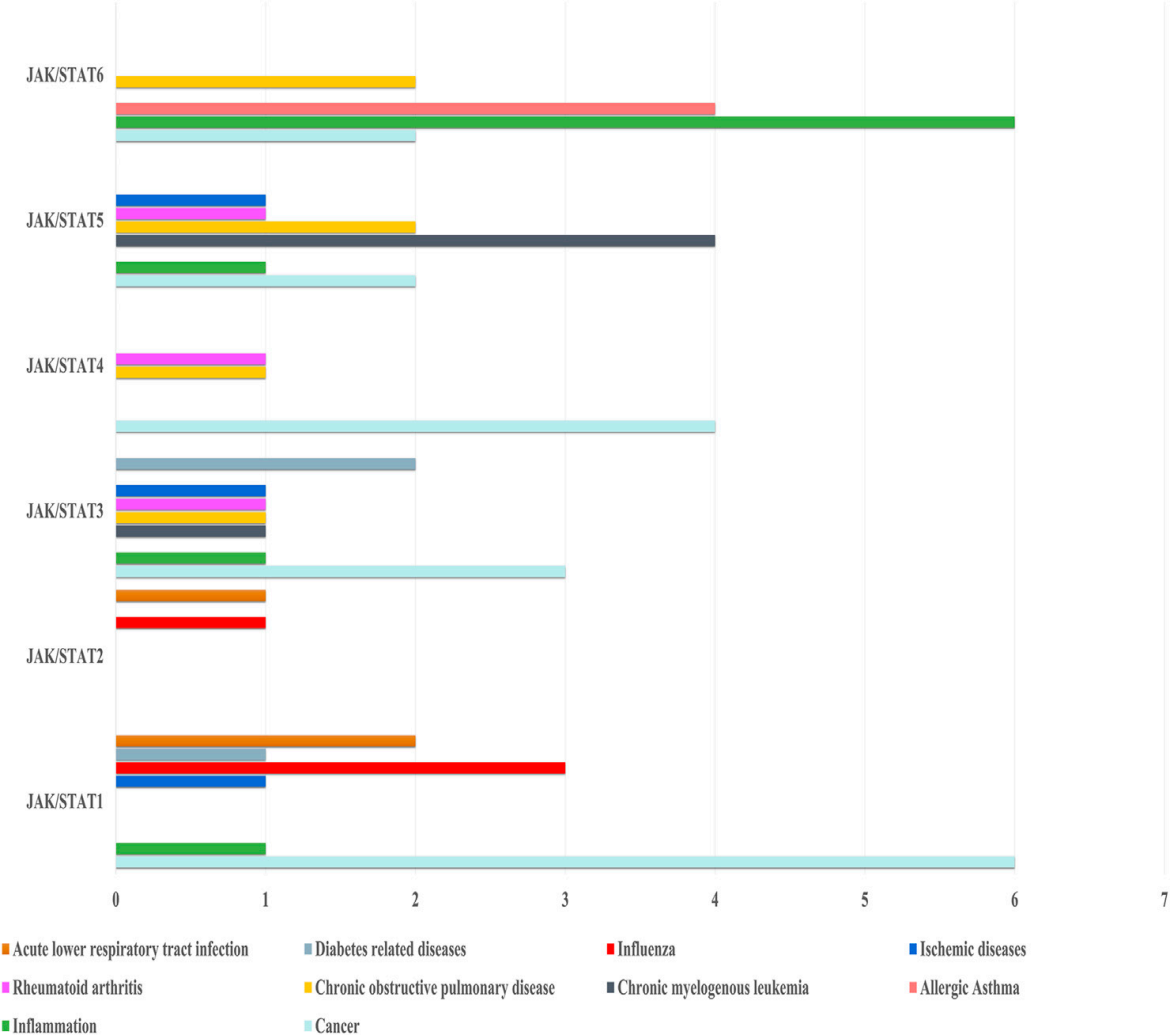
TCM formulas and their corresponding extracts or active compounds in the treatment of diseases through regulating the JAK/STAT signaling pathway.

Chinese herbs in traditional Chinese medicine (TCM), which have widespread distribution throughout China, are the gold resources of China and have been extensively used for treating multiple diseases for thousands of years. However, Chinese herbs and herb formulas exert their effect on treating diverse diseases through JAK/STAT pathways that have not been elaborated, which limits their approval and applications. In this review, we summarized various herbs, herb formulas, natural compounds, and phytochemicals isolated from herbs that have the potential for regulating multiple biological processes *via* modulation of the JAK/STAT signaling pathway based on the published work. Regarding natural compounds, for instance, celastrol alleviated hypertensive heart disease and cancer metastasis through inhibiting STAT3 (Ye et al., 2020) and STAT6 (Yang et al., 2018) signaling, respectively. Luteolin helped treat acute lower respiratory tract infection by activating STAT1 (Wang et al., 2020a) and alleviating inflammation through both inhibiting STAT3 and activating STAT6 (Wang et al., 2020b). Cryptotanshinone was regarded as the STAT3 and STAT5 regulator for ischemic stroke (Zhu et al., 2021) and chronic myelocytic leukemia (Dong et al., 2018). Baicalin regulated multiple members of the STAT family, including STAT1, STAT3, STAT4, and STAT5, for treating T2D-induced liver tumor, sepsis, and myocardial ischemia reperfusion injury (Jiang et al., 2022). In addition, dihydroartemisinin ameliorated multiple sclerosis (Du et al., 2021a) and gastric cancer (Liang et al., 2021a) through regulating STAT1 signaling. Moreover, Yu-Ping-Feng decoction (Boriero et al., 2021; Zhou et al., 2021), Sijunzi decoction (Xiong and Qian, 2013), and Yu-Ping-Feng powder (Xiong and Qian, 2013) exerted their effects on anti-NSCLC and spleen deficiency through influencing STAT1 signaling. Meanwhile, we also summarized the candidates for treating diverse diseases involved in JAK/STAT signaling, such as anti-cancer, ameliorating inflammation, allergic asthma, chronic kidney disease, rheumatoid arthritis, and fibrosis. Regarding the aspects of antineoplastics, eight compounds from natural plants or herbs were collected, namely, lanatoside C, saikosaponin A, celastrol, cynaropicrin, dihydroartemisinin, imperatorin, baicalin, and silibinin, which exhibited their potential for anti-tumor or anti-cancer effects. Moreover, inhibitory effects of STAT1, STAT3, and STAT4 activation by these compounds still come to predominate, which was the same as that regulated by two formulas and two extracts. In this respect of anti-inflammation, including airway inflammation, RA, and arterial inflammation, these TCM and their corresponding extracts or active compounds are preferred to influence STAT1 and STAT6 signaling, which are shown in Figure 2. These extracts or active compounds belong to heat-clearing (Qingre) and dewetting (Zaoshi), heat-killed (Xiehuo), and detoxifying (Jiedu) traditional Chinese medicines and are used for treating inflammation and cancer in clinical formulas by regulating JAK/STAT alone, or its crosstalk with NF-κB, and Notch signaling.

Overall, our study will provide support for revealing TCM formulas and their corresponding extracts or active compounds in the treatment of diseases and the underlying mechanisms, which further improve rapid TCM spread to the world and drug discovery regarding regulating STAT signaling.
